# The Only Woman in the Room: Oral Histories of Senior Women Physicians in a Midwestern City

**DOI:** 10.1089/whr.2020.0041

**Published:** 2020-08-24

**Authors:** Anne Walling, Kari Nilsen, Kimberly J. Templeton

**Affiliations:** ^1^ Department of Family and Community Medicine, University of Kansas School of Medicine-Wichita, Wichita, Kansas, USA.; ^2^ Department of Orthopedic Surgery, University of Kansas School of Medicine-Kansas City, Kansas City, Kansas, USA.

**Keywords:** gender equity, medical education, older female physicians, senior physicians

## Abstract

***Introduction:*** The female students of the 1960s and 1970s have been at the forefront of issues for women in medicine throughout their careers. They have personally experienced the diverse challenges and opportunities that have continued to arise, for women in medicine over the past 50 years. Capturing their stories can provide a unique contribution to the history of women in medicine, especially in documenting the crucial transitional decades during which women entered the profession in increasing numbers. Their experiences can also inform programs to improve the careers of current and future women in medicine.

***Materials and Methods:*** We partnered with the Medical Society of Sedgwick County to invite all women who had been members before 1990 and still lived in the area to participate in focus groups about their experiences in medical school and residency. Interviews were recorded, and the recorded discussions and field notes were analyzed by using a thematic analysis approach

***Results:*** Discussions revolved around several topics, including motivations to become a physician, family attitudes, experiences during medical school and residency, and experiences with co-workers and patients. Illustrative quotes were selected for the themes identified.

***Discussion:*** This project illuminates the motivations, attitudes, and experiences of a diverse group of women who entered medical school in the 1960s and 1970s. Although they came from very different backgrounds and trained in a variety of institutions and specialties, their stories revealed consistent themes, many of which remain relevant for female physicians.

***Conclusion:*** This unique cohort of women were part of the major transition from times when women were rare in medicine to being at least half of physician trainees. Their experiences should be used to inform the profession moving forward.

## Introduction

Remembering what it took us to get here will, I hope, help to ensure the equal success of future generations of physicians independent of their sex^1^

**FIG. 1.**
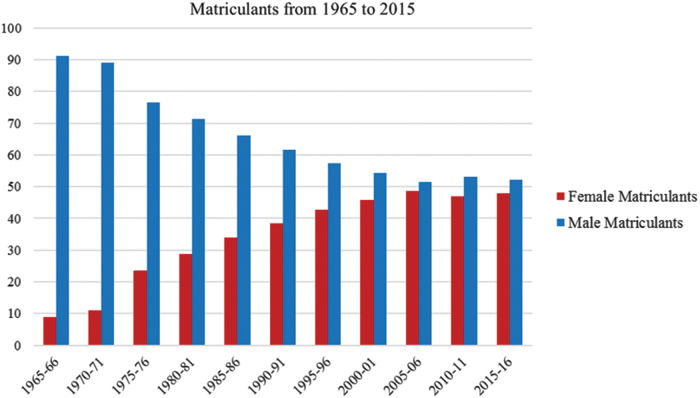
Percentage of female and male first year U.S. medical students. Prepared with data from: Association of American Medical Colleges (2016).^2^

In 1965, fewer than 10% of medical school matriculants were women: By 1975, this had risen to just more than 20% (Fig. 1).^2^ This was not the first period in which the number of women entering medicine increased. During the 1890s, women accounted for more than 30% of graduates from some medical schools, and, although excluded from others, also had the option of attending 1 of 17 all-female medical colleges.^3^ By 1910, the United States had an estimated 9015 female physician, representing 6.0% of the profession. For complex and synergistic reasons, progress stalled, and the percentage of women physicians remained around 4.4%–6.1% for the next 50 years.^3^ The female medical students of the 1960s and 1970s grew up in a world where medicine was almost exclusively a male profession. They faced significant discouragement and many challenges in making the unusual and often controversial decision to enter medicine.^3–8^ In medical school, they were a minority in a “hostile environment.”^9^ In 1965, >85% of U.S. medical schools reported that fewer than 10% of students were women.^10^ Female medical students faced internal challenges over role conflict, stress, and anxiety as well as the isolation, resentment, harassment, and other institutional challenges that are well documented in contemporary reports.^3,6,9–17^

As the first large wave of women in medicine in the 20th century, the female students of the 1960s and 1970s have been at the forefront of issues for women in medicine throughout their careers They have personally experienced the diverse challenges and opportunities that arose, and continue to arise, for women entering male-dominated professions over the past 50 years. Capturing their stories provides unique insights into the watershed decade when women reached a “tipping point” in U.S. medicine.^1,3^

Oral histories from participants complement and enrich the written record of significant events and improve the understanding of crucial periods.^18^ The few available oral histories of women in 20th-century medicine focus on individuals with the most distinguished careers.^19–21^ We sought to collect recollections from a diverse group of women physicians who entered medical school before 1975 and participated in the first wave of the current transition of the profession toward greater gender equity.

## Materials and Methods

### Environment and participants

We partnered with the Medical Society of Sedgwick County (MSSC) to identify female physicians older than 60 years of age. Almost all physicians practicing in Wichita, KS, have been MSSC members since its founding in 1903. Although the earliest record of a female physician in Wichita was in 1875, by the late 1960s, the MSSC only had 16 to 18 female members—around 4% of the total. In 2019, the 280 female MSSC members represented about 30% of the membership.

### Focus groups

In Fall 2018, we invited all women who had been MSSC members before 1990 and still lived in the area to participate in focus groups in Fall 2018 regarding their experiences in medical school and residency. Lunch was provided. Participants signed informed consent forms, and the study was approved by the University of Kansas Institutional Review Board.

The discussions were facilitated by members of the research team and they contained around eight questions (Table 1). Sessions were audio recorded, and two research team members took notes during the sessions. As saturation of themes was apparent, only two sessions were held.

**Table 1.** Prompt Questions for Focus Groups

Why did you become a physician? What motivated you?
How did your family feel about you deciding to become a physician?
What experiences in high school helped or hindered you in pursuing a career in medicine?
What was college like for a woman interested in medicine?
Tell us about the admissions process. What were you asked?
Tell us about your experiences as a medical student. How were you treated by:
- the faculty and other teachers
- nurses and staff
- male medical students
- patients and others?
How was your experience of getting into a residency program?
Tell us about your experiences as a resident.
Overall, what are your thoughts on your medical education and being a female physician?

### Analysis

The recorded discussions and field notes were analyzed independently by two researchers (K.N., A.W.) using a thematic analysis approach. Thematic analysis follows an inductive process of becoming familiar with the data, generating initial codes, and finally identifying and refining common patterns or themes across qualitative data.^22^ Both investigators independently coded the focus group recordings and reached consensus on an agreed coding framework. The principal researcher (A.W.) then completed the remaining coding and recursively refined a thematic structure in discussion with the second author (K.N.). Patterns of commonality and divergent views were identified. The researcher team compared and discussed findings to develop the final themes by consensus. These findings were returned to the participants for validation.

## Results

All 23 participants were aged 60 years or older and entered medical school between 1965 and 1975. Most attended medical schools and/or residency programs in the Midwest, but individuals had graduated from institutions across the nation. Participants represented nine medical specialties, that is, family medicine, internal medicine, neurology, obstetrics/gynecology, ophthalmology, pediatrics, psychiatry, radiology, and surgery. Nine (39%) were in full-time practice, nine (38%) were in part-time practice, and five (22%) were retired. Three were Hispanic, and 20 were White. All were currently or previously married.

### The decision to become a physician

#### Motivations

All participants grew up in environments where medicine was almost exclusively a male profession. A few of the older participants mentioned female physician role models. One recalled being inspired as a child in the 1950s by a woman surgeon: “She told me about her daughters. I thought it was so cool to take my tonsils out then go home and be a mommy.” Another participant recalled receiving care through recurrent childhood illnesses from an “adored woman GP.” The younger participants named several of the older members of the group as “trailblazers and role models,” revelations that surprised and embarrassed the older women.

Most of the group was strongly motivated by interest in the scientific and technical aspects of medicine. All expressed a “sense of vocation, being called to medicine” and were motivated by the opportunity to do useful, challenging work. “It just seemed the right thing for me to do—to be able to make a difference.” For some, the motivation was spiritual: “I was inspired by a book about a medical missionary.” Others recounted family experiences with illness and admiration for the care provided by the medical team: “my cousin died of leukemia—I decided to be a doctor aged seven.”

Many participants, especially the older physicians, had been “expected and guided into traditionally female roles, especially nursing or health professions.” Several had been in training or qualified in other health professions before entering medicine. These women described frustrations with the hierarchical structure and limited roles of nonphysicians in health care as strong motivations to becoming physicians. One former nurse commented, “I realized I was at the wrong end of the chain of command. I was frustrated by the power structure and thought, ‘I'm as smart as they (physicians) are.’” Others were motivated by supportive physicians: “I was a CNA and surgeons invited me in to see surgeries: I got hooked on surgery.”

Almost all participants clearly recalled coming to the realization that they could aspire to be a physician despite discouragement. Many identified specific events or episodes “when it suddenly hit me that I could do that”; others described the growing recognition that they were as well or better qualified than their male classmates who were being steered toward medicine. Most of the group recounted thinking, “I am as good/better than they are!!” For some, the prestige of medicine was an incentive. “It was the hardest thing to get into so only the smart kids did it: I did it for pure snob ‘wow’ value.”

### Family attitudes

The most common parental and family attitudes were summarized as “supportive but concerned. They weren't worried about me managing the work but thought I was setting myself up for a lot of challenges and hassles.” Fathers were frequently described as supportive and proud of a daughter's aspiration to become a physician: “Go for it. You can do anything you put your mind to. Aim high.” The attitudes of mothers were mixed, and they ranged from strong support and validation, especially from mothers who had been denied career opportunities themselves, to attempts to sabotage the decision. “My mother cried because she thought it meant I would never get married. Mother said I was just showing off and would never get in.” Some families were not supportive or even opposed a girl going to medical school: “my family thought I was crazy.” Sometimes, individual family members were very influential: “My mother and grandmother were arguing with me, then my great-uncle (the patriarch) announced I should go, and he would pay for the books!”

Participants came from a wide range of backgrounds and several were the first women in their families to attend college. For some of these women, families expressed unease about being able to “fit in” with students from more prosperous and educated families. Many families were unable to financially support their daughter's medical education. Several participants “had to find a way to pay for it all myself.” One participant described encouragement but very little financial support from her mother, a widowed mother of six children. Several participants expressed deep appreciation of the concern and expense incurred by their families: “I don't think I realized at the time how much they worried about me.”

### High school and college experiences

In high school and college during the 1960s, the participants routinely encountered discouraging and disapproving attitudes toward their aspirations to become physicians. Most teachers and advisors expressed “surprise and somewhat patronizing discouragement. It just wasn't thought about for girls. I was in a convent school and medicine was just not considered ‘nice’ or suitable for a good Catholic girl.” Discouragement ranged from passive amusement to active antagonism and denial of opportunities. “Even when I was top in the class, I was not put forward for a medical career or advancement. I was coaching the guys in my class in science and they got put in for SATs and I didn't.” In high school, many participants were “often the only girl in science classes.” Schools routinely offered fewer opportunities for girls; “our school didn't even have girls' sports!” A common teacher attitude encountered by the older participants was “No point in teaching this to a girl.” Only a few of the older physicians recalled supportive teachers or advisors in school. Support sometimes came from unexpected quarters. One remembered being encouraged by a Latin teacher to persist with her goal of medicine. Conversely, during career day, another participant was told publicly by her much-admired family physician that “women have no place going into medicine.” The younger members of the group had more supportive high school experiences. One reported, “I was valedictorian so it was expected I would become a doctor and return to my small town to practice.”

In college, several participants reported difficulties in securing the required science courses and encountering negative attitudes, even overt hostility, in these courses. “The professor told me I couldn't get into the class because ‘Girls don't understand physics.’” “The TA told me it was a ‘waste his time teaching a girl.’” Those who persisted often faced being taunted, or conversely ignored, in class. “They just ignored me and would not answer my questions—it was like I wasn't there.” Most recalled being expected to do menial tasks such as taking notes in meetings, making coffee, or cleaning laboratory equipment. “I was the only girl: I was assigned clean up jobs in graduate school.” The women also experienced social challenges, including disapproval and/or becoming alienated from female friends. “It was not cool to be good at science in college: all my friends were flunking out of physics.” Some of the older groups commented, “no guys wanted to date pre-med and we were too busy anyway!”

A few of the older participants were in relationships on entering medical school but this was more common for those who entered medical school later. The importance of a “supportive husband or partner” was frequently repeated throughout the discussions.

### The admissions process

The participants who applied to medical school in the 1960s described the norm; “of course, there were quotas for females—about 10% of class—but it changed very rapidly in the 1970s. They told me they would only take 10 girls; so, I was determined to be one of those girls!! It was just the way it was.”

All participants encountered patronizing attitudes during interviews. Everyone was asked, “why not be a nurse?” They all were challenged about “taking a man's place in medicine,” said that women “couldn't handle the work in medical school,” and that “women physicians don't work as hard as men.” Some of these attitudes were perceived as genuine beliefs that a woman was a “poor value for the investment in a medical education, a wasted place in medical school.” Interviewers sometimes appeared concerned for female applicants; “they didn't think I had the stamina for it; they told me ‘it gets pretty rough.’” One participant recalled being told she was “too pretty to be getting into this.” All participants, including those who were interviewed later in the 1970s, recalled consistently being questioned about current relationships and plans for marriage and children. Those in relationships recalled regularly being asked “what my husband/boyfriend thought about me doing medicine.”

Overt hostility to women in medicine was commonly encountered, especially by those who applied to medical school in the 1960s. One participant described walking out of an interview because of hostile questioning and being “begged to come back.” Some schools required aptitude tests. “They had different aptitude for medicine tests for females and males. I did both and scored better on the male test!”

In contrast to faculty interviewers, several women had positive experiences with admissions office staff and recounted examples of individuals—all older women—going to great lengths to help them navigate the process. “I was late applying, and one lady literally walked me over to different offices to get everything done. I did not think I could afford it, but a lady told me all about scholarships and helped me do the paperwork. I could only be an MD because I got a scholarship. I didn't know what I was doing but the admissions office staff just showed me what to do. They were really encouraging.”

### Experiences during medical school

Most participants entered medical school in the early 1970s, with the earliest in 1965. The earlier group reported “feeling very lonely” and that “it was important to know about guy things, like football.” Those entering after the mid-1970s reported: “It was more normal to have girls in the class.” These women reported similar experiences of gender-based harassment and discrimination but to a lesser degree than the older group. They also felt less isolated. All participants described the paradoxical situation of being both “more exposed and invisible or ignored.”

The faculty members were almost exclusively male. “We had two female faculty members in the entire medical school, and they were tough old bi***hes—worse than the men.” All participants reported a general experience of being held to higher standard than their male classmates. “You were more visible, more exposed. Girls had to work harder, you had to know your stuff.” Some of the older participants suspected that they had been discriminated against in grading and award of prizes,

Overall, faculty attitudes were “somewhat neutral with glaring exceptions.” Comments reported included, “what are YOU doing here; why are you taking a man's place? I'm not going to bother with you as you will drop out.” Women regularly experienced humiliation and intimidation in class. Sexist attitudes, comments, and jokes were common, including use of pornographic slides and handout materials. Women felt taunted by crude or sexist language and jokes and were expected to at least tolerate inappropriate language and behavior. “Can't you take a joke? Don't be a prude. I felt constantly caught between being a ‘ bi**h’ and a ‘cutie.’” Several participants recalled “faculty and residents who really wanted to make you cry or blush on rounds in front of everybody.” Everyone regularly experienced *being called* “honey,” “sweetie” or “things like that—by patients as well as faculty and others.” The older women spoke of female classmates who dropped out because of mistreatment: the “vulnerable ones had like a target on their backs; they were picked off by the victimization.”

Physical harassment was more common and more severe for the older women but was experienced by all group members to some degree. “One professor would stand right over me and get right in my face.” Uncomfortable physical proximity was common. Several reported inappropriate touching and overt sexual harassment. “The OB-GYN department was ‘the WORST’ for overt misogamy.” None of the group recalled taking any action to address sexual harassment. “You learned who the gropers were and tried to avoid them.”

Male classmates were generally supportive. “Few of them were hostile or disturbed by women in class.” They never acted to challenge or prevent inappropriate behaviors by faculty or staff (especially nursing) toward female students but did intervene when other students acted inappropriately. “The overt bullying and hassle by a couple of guys was negated by the other male students. One guy exposed himself and taunted me; the others intervened and made it VERY clear that I was to be respected.”

Female medical students “always had to dress professionally.” For the older group this required “suits, skirts and heels, plus hose; NO trousers, and skirts had to cover your knees. This was the ‘60s—I ONLY owned mini-skirts and had to buy ‘granny skirts’ to wear in the hospital.” For surgery, they were “not allowed to wear scrub pants—we had to find surgical scrub dresses, but the nurses wouldn't give us any.” Many experienced hostilities from surgical and obstetric nurses and were “banned from both the nurses' and physicians' changing rooms. We changed in a closet.” Others recalled changing in the bathrooms or even “wearing surgical scrubs under my clothes and stripping off in the corridor.”

Sleeping arrangements and “on call” rooms presented major challenges. “The resident told me his wife would not let me sleep in the same room as him: I had to sleep on a gurney in the hall. We slept four to a room in bunk beds when on call. Some of the guys were uncomfortable with me there but I just joked that I wouldn't jump on them during the night.”

### Experiences during residency

For many of the older women physicians, securing a residency position was more difficult than getting into medical school. The younger group still found this challenging, but by then female applicants were more common in several specialties. Sexist attitudes were commonly encountered during the interview process, especially around comments about having enough stamina to fulfill all the necessary duties. Some women reported interviewers who questioned whether women could function in certain specialties, especially surgery, “women have no place in the operating room.” Sometimes, these comments were expressed as doubts that a woman could earn the respect and be able to appropriately manage patients, staff, students, or other residents. Although the Civil Rights Act was passed in 1964, all participants were regularly asked illegal and inappropriate questions during interviews, including about personal relationships and plans for pregnancy. Interviewers commonly told the younger women, “we've had problems when females get pregnant and we must do their work.” Once accepted, several participants felt they were the “token female resident” and one recalled the department chairman boasting about having a female resident “to show how progressive the program was.” Conversely, one participant was told by the head of the unit that her appointment “was a huge mistake by the Administration to look good.”

Many of the issues encountered during medical school persisted into residency. Again, the older group had more frequent and severe problems. Issues over dress, changing rooms, and on call sleeping arrangements were major problems for the older women during residency. In one case, a senior professor had to intervene to force the institution to provide a changing area for female medical students and residents.

Faculty and colleagues were generally more supportive during residency, but the older male physicians were frequently patronizing. Many women were denied career opportunities. “They assumed I was not serious about my career, so they didn't even consider offering me fellowship.” This was particularly noticeable for the married women: “they assumed I would quit practice or maybe do something part-time. My husband was a physician and I was told by the program director's wife that my ‘vocation was to be a doctor's wife and support my poor husband!’”

Relationships with nurses were often problematic. Older nurses told me, “this is not what you should be doing.” “They were obstructive, rude, and very difficult to work with. It was hard for nurses to take orders from a female. The guys could yell but I learned women must never yell at nurses.” Conversely younger nurses “were not pleased to see a woman as they wanted to date the residents and marry a doctor.”

The women also experienced issues with patients. “Throughout med school and into practice, patients and others did not expect woman physician—I was regularly mistaken for a nurse or told to go and get the real doctor.” Some patients had inappropriate attitudes or made offensive remarks about female physicians. Conversely, some patients expected woman to have deep “understanding” or be more caring and sympathetic than male colleagues. “Some patients avoided seeing me because I was a female and some sought me out.” Those in surgery found their practice skewed, even in residency, toward female conditions such as breast disease.

The older women physicians encountered many problems over pregnancy. “They just did not know what to do with a pregnant resident. Everyone was very uncomfortable. There were no policies and I was not ill so I couldn't use sick leave. This meant time off to have the baby had to be vacation or I was in breach of my contract.” Another woman was told by an unmarried older female physician that patients, staff, and colleagues were “distressed to see you pregnant” and that her pregnant appearance was “uncomfortable for patients.” One attending physician told a participant, “residents shouldn't have time to get pregnant!”

### Reflections on medicine as a career for women

All participants expressed great satisfaction and sense of fulfillment from their careers. “It's been a wonderful experience and I would not do anything else. What else could you do that would give you such opportunities?” When prompted to identify regrets, discussion focused on lack of personal time, especially to build friendships and collegial networks. “There was nothing left over between work and family, so we never got to know other women doctors—and there were not a lot around.” Especially in the older group, the women still feel more comfortable in male company because “we were socialized as guys.”

All participants enthusiastically endorsed medicine as a “wonderful career for women.” They supported the increasing numbers of women in the profession, especially in specialties and leadership roles that had traditionally been male dominated. They recognized that their experiences were determined by the time and unique circumstances of their training and may be of limited use to women currently entering or progressing in the profession. “Things are different now—they will still face problems, but they may be different or less obvious than when we were students. They can still find themselves as the only woman in the room. I just worry that the girls will think all the bad stuff has been fixed and that they will have an easy time.” These women described having to work out how to function in medicine at every step of their training—a situation that they have continued to encounter throughout their careers. “It was like, they said we could come into medicine but they did not think it through or make any preparations.” They repeatedly stressed that their stories of unfair or even cruel experiences should not encourage a sense of entitlement in younger women in medicine.

## Discussion

This project illuminates the motivations, attitudes, and experiences of a diverse group of women who entered medical school in the 1960s and 1970s. After some initial reticence, “I didn't think we were doing anything special,” and participants shared a rich trove of stories from a wide range of experiences. Although they came from very different backgrounds and trained in a variety of institutions and specialties, their stories revealed consistent themes. Participants who matriculated after about 1974 reported identical themes to the older women but described fewer and less overt gender-based issues, presumably due to increasing numbers of women in the class plus the contemporary social changes and federal initiatives (such as Title IX regulations).^3^

All participants were attracted to medicine for altruistic reasons and encountered obstacles, discouragement, and even mockery in their efforts to become physicians. They met diverse challenges with determination, tenacity, and hard work. Challenges ranged from extremely serious issues such as physical abuse and denial of career opportunities to practical problems such as finding scrub dresses. They lacked mentors and support systems to encourage them to enter medicine and to support them during training. Anticipated sources of encouragement and help, such as teachers, nurses, and female friends, were often disappointing, but support was sometimes found in unanticipated places, such as staff of the medical school admissions office.

Throughout medical training, these women dealt with discrimination, harassment, intimidation, bullying, denial of opportunities, and other forms of unfair treatment; yet they told their stories with humor, forgiveness, and reconciliation. They expressed some sadness, especially over denied opportunities or acknowledgments, but had little bitterness or appetite for revenge. Many wished they had handled specific events differently, but group members expressed few regrets. They told stories that current trainees might find horrific and almost incredible, but the group was very positive, energetic, joyful, and resilient.

The study findings are limited by the small number of participants and lack of diversity. Identification of eligible participants relied on knowledge of the medical community, as dates of birth and medical school graduation were not recorded in the MSSC membership database. Scheduling was challenging as potential participants, including the retirees, had very busy schedules. Recall of events from >40 years ago could be selective or inaccurate. The general attitude was very positive, and participants may have avoided sharing distressing or deeply personal experiences, particularly regarding abuse or incidents involving individuals in the local medical community.

## Conclusion

This unique cohort of women participated in the initial stages of the major transition that took women from being rare in medicine to accounting for more than half of physician trainees. It must be stressed that these are the stories of the survivors and they are told by successful physicians who felt comfortable discussing their experiences. All participants knew of women medical students or physicians who had suffered significantly, even committing suicide. A more complete view of the experience for women students of the period would include women who did not complete training, dropped out of medical practice, or did not feel comfortable participating in the groups. In particular, the experiences of women who left medicine, might identify modifiable factors behind their loss to the profession.

Despite significant progress, many of the concerns reported by these older female physicians are still valid. Many female physicians lack time to develop supportive networks, struggle to balance work and personal responsibilities, and are vulnerable to burnout and its attendant morbidities.^23^ Women physicians continue to experience gender-based discrimination, sexual harassment, salary inequity, and denial or limitation of advancement opportunities.^23^ Much remains to be done, and it can never be taken for granted that positive changes in the culture of medicine will continue.

The medical students of the 1960s and 1970s helped shape the modern environment of medicine for women. Understanding their experiences can identify pitfalls and may inform how to sustain and build on the positive momentum of the past 50 years.

## Abbreviation Used

MSSC: Medical Society of Sedgwick County
